# Simultaneous Retroperitoneal Robotic Partial Nephrectomy and Hepatectomy for Synchronous Renal-Cell Carcinoma and Hepatocellular Carcinoma in a Cirrhotic Patient

**DOI:** 10.1089/cren.2016.0096

**Published:** 2016-11-01

**Authors:** Khaa Hoo Ong, Steven Kuan-Hua Huang, Chia-Sheng Yen, Yu-Feng Tian, Ding-Ping Sun

**Affiliations:** ^1^ Division of Gastroenterology & General Surgery, Department of Surgery, Chi Mei Medical Center, Tainan, Taiwan.; ^2^ Division of Urological Oncology, Department of Surgery, Chi Mei Medical Center, Tainan, Taiwan.; ^3^ Chai Nan University of Pharmacy and Science, Tainan, Taiwan.

**Keywords:** retroperitoneal, simultaneous, robotic surgery, hepatectomy, partial nephrectomy

## Abstract

***Background:*** The development of laparoscopic and robotic surgeries represents the modern era with the objective of improving patient outcomes; this surgical method is widespread in urology and general surgery. Retroperitoneal laparoscopic/robotic surgery is common in urologic surgery, but not in liver surgery. Tumors located in the posterosuperior aspect of the liver are difficult to access using a transperitoneal approach, and control of bleeding can also be difficult, especially in patients with cirrhosis.

***Case Presentation:*** Herein, we present a 66-year-old man who had a cirrhotic liver with concurrent renal and hepatic tumors. The renal tumor was located at the upper pole of the right kidney and the liver tumor was located at the liver dome (segment VII); the patient underwent simultaneous robotic hepatectomy and partial nephrectomy with a retroperitoneal approach.

***Conclusion:*** To our knowledge, this is the first case involving a retroperitoneal approach for a simultaneous robotic hepatectomy and partial nephrectomy; this method was feasible and safe. We hope this approach serves as an alternative surgical method for patients with synchronous renal and posterior segment liver tumors.

## Introduction and Background

The development of laparoscopic and robotic surgeries represents the modern era with the objective of improving patient outcomes and surgical morbidity; this modality is widespread in urology and general surgery. Retroperitoneal laparoscopic and robotic surgeries are common for the urologists, but are rare approaches by liver surgeons.

Many studies have demonstrated the benefits of laparoscopic/robotic liver resection, including smaller incisions, less estimated blood loss, less packed red blood cell transfusions, decreased narcotic requirement, shorter length of stay, and diminished postoperative morbidity when compared with open hepatic resection.^1,2^ However, tumor size and location influence procedural difficulty, that is, tumors located in the right posterosuperior sector of the liver can be difficult to approach because of poor observation, and instruments can become difficult to dislodge because of the large liver volume anterior to a tumor during transperitoneal laparoscopic/robotic surgery. Thus, a retroperitoneal laparoscopic/robotic liver resection may be a feasible technique for posterosuperior sector tumors, especially in patients who undergo combined surgery performed by a urologist.

## Presentation of Case

**FIG. 1.**
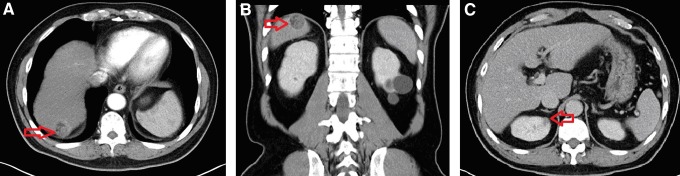
**(A)** Liver CT axial view: A 2 cm hypervascular tumor (*arrow*) was located at the S7 liver dome. **(B)** Liver CT coronary view: The tumor was located at the dorsal aspect of the liver dome. **(C)** A 1 cm renal tumor was located at the upper pole of the right kidney; we suspected that the tumor was renal-cell carcinoma.

A 66-year-old man has a history of chronic hepatitis B, hypertension, and coronary artery disease controlled by perindopril, 4 mg once daily, and clopidogrel, 75 mg once daily. He had a regular annual physical examination in our hospital, and incidentally, a 3 cm hypervascular tumor was observed at the S7 liver dome in an abdominal computed tomography scan; hepatocellular carcinoma (HCC) with liver cirrhosis was suspected (Fig. 1A, B). He had another 1 cm exophytic tumor lesion that appeared to be renal-cell carcinoma (RCC) at the upper pole of the right kidney (Fig. 1C). Creatinine level was 0.6, α-fetoprotein value was 6.1 ng/mL, and the Child–Pugh score was A. He denied any symptoms and had normal findings upon physical examination.

Retroperitoneal Da Vinci robot-assisted simultaneous hepatectomy and partial nephrectomy were performed. Under general anesthesia with endotracheal tube intubation, the patient was placed in a left decubitus position with flank banding.

**FIG. 2.**
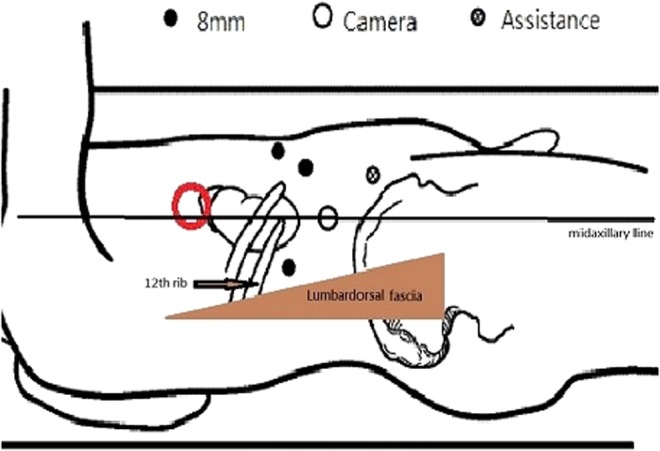
Trocar position: The camera port was placed at the midaxillary line of the midpoint between the 12th rib and iliac crest. The second robotic arm port was placed at the triangular area of the lumbodorsal fascia and 12th rib; the first and third robotic arm ports were placed at the right subcostal margin with an 8 cm distance between each port. An accessory port was placed at the right lower quadrant of the abdominal wall that depended on the tumor location.

Retroperitoneal access was obtained with a balloon dilator, and the first trocar was placed. We inserted a 0° laparoscope, and the retroperitoneal space was expanded with the laparoscope to dissect the fat plane. The port was connected to a CO_2_ insufflator with the pressure set at 14 mm Hg. Three robotic and one assistant working ports were inserted under endoscopic monitoring and finger guidance (Fig. 2).

After exposing the lower pole of the right kidney the renal artery was identified and controlled with a Bulldog clip. The tumor was located using intracorporeal sonography, and the renal mass was cut with a pair of scissors. The renal defect was closed with 3-0 Monocryl continuous suture and the surface was covered with the Surgicel for hemostasis. The total amount of ischemic time was 19 minutes.

**FIG. 3.**
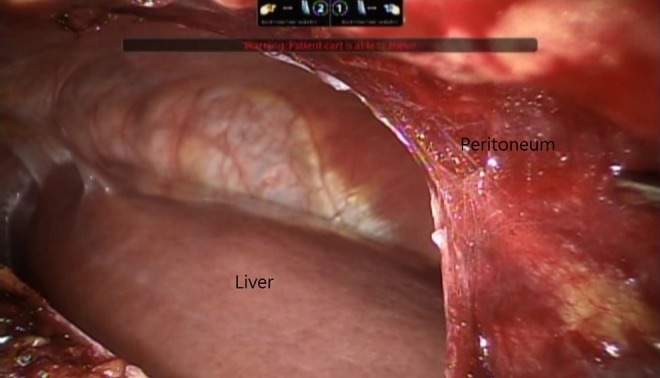
The peritoneum was thin, and the liver could be clearly identified posteriorly; this position could reduce the risk of transverse or ascending colon injury.

With the same docked Da Vinci system, the peritoneal layer was identified and incised after confirming with direct vision that no visceral organ was behind it (Fig. 3). Pneumoperitoneum was performed using the same pressure (14 mm Hg); the right lobe of the liver was mobilized after triangular ligament and coronary ligament were lysed. While the patient was in a left decubitus position, the tumor located at the S7 liver dome was moved to the center of the operator's working space. The liver tumor was identified using sonography, and a partial hepatectomy was performed using a Harmonic scalpel without inflow control. After completing the partial hepatectomy, both specimens were removed through a small incision in the right flank, and a vacuum ball drain was placed. The total blood loss was 200 mL. Postoperative pathology analysis confirmed the diagnosis of RCC and HCC with negative margins; the normal liver portion had definite cirrhosis with an Ishak score of 6/6. The patient recovered well, and he was discharged on postoperative day 5.

## Discussion

The first retroperitoneal laparoscopic hepatectomy was reported in 2011 that was performed for a 2 cm metastatic colorectal cancer tumor located at segment VI.^3^ In 2015, Yin and colleagues provide a case report and video of a laparoscopic retroperitoneal hepatectomy for a small 2 cm HCC at segment VI in a noncirrhotic liver.^4^ Both demonstrated that a retroperitoneal approach is possible for liver resection. We presented a case involving a simultaneous retroperitoneal robotic partial nephrectomy and hepatectomy for RCC in the right kidney and HCC in the liver dome (segment VII) of a cirrhotic patient. In a left decubitus position, the tumor could be moved into a more convenient working space and increase the ease of performing a partial hepatectomy, even for posterosuperior sector liver tumors. A retroperitoneal method can also reduce the risk of visceral organ injury, as compared with a transperitoneal laparoscopic hepatectomy performed with patients in a left decubitus position because we could recognize the visceral organs behind the thin layer of peritoneum before we incised it.

## Conclusion

A retroperitoneal laparoscopic/robotic hepatectomy appears to be an excellent technical approach for tumors located superficially in the right posterior hepatic segment, even at the dome of the liver, especially in patients with a cirrhotic liver who would not be a candidate for a major liver resection. To our knowledge, this is the first case of a simultaneous retroperitoneal robotic partial nephrectomy and hepatectomy; this approach is feasible and safe for resection of liver tumors in the posterosuperior sectors. We hope that this alterative surgical method can be used for patients with synchronous renal and posterosuperior sector liver tumors and that a liver surgeon can perform this method through the retroperitoneal space.

## Consent

A written informed consent from the patient for publishing the case report and images is available for review by the editor of this journal.

## Abbreviations Used

CT: computed tomography
HCC: hepatocellular carcinoma
RCC: renal-cell carcinoma
